# Enterovirus 71 3C Protease Cleaves a Novel Target CstF-64 and Inhibits Cellular Polyadenylation

**DOI:** 10.1371/journal.ppat.1000593

**Published:** 2009-09-25

**Authors:** Kuo-Feng Weng, Mei-Ling Li, Chuan-Tien Hung, Shin-Ru Shih

**Affiliations:** 1 Research Center for Emerging Viral Infections, Chang Gung University, Kwei-Shan Tao-Yuan, Taiwan, R.O.C.; 2 Department of Medical Biotechnology and Laboratory Science, Chang Gung University, Kwei-Shan Tao-Yuan, Taiwan, R.O.C.; 3 Graduate Institute of Biomedical Sciences, Chang Gung University, Kwei-Shan Tao-Yuan,Taiwan, R.O.C.; 4 Department of Molecular Genetics, Microbiology and Immunology, UMDNJ-Robert Wood Johnson Medical School, Piscataway, New Jersey, United States of America; Centro de Biología Molecular Severo Ochoa (CSIC-UAM), Spain

## Abstract

Identification of novel cellular proteins as substrates to viral proteases would provide a new insight into the mechanism of cell–virus interplay. Eight nuclear proteins as potential targets for enterovirus 71 (EV71) 3C protease (3C^pro^) cleavages were identified by 2D electrophoresis and MALDI-TOF analysis. Of these proteins, CstF-64, which is a critical factor for 3′ pre-mRNA processing in a cell nucleus, was selected for further study. A time-course study to monitor the expression levels of CstF-64 in EV71-infected cells also revealed that the reduction of CstF-64 during virus infection was correlated with the production of viral 3C^pro^. CstF-64 was cleaved *in vitro* by 3C^pro^ but neither by mutant 3C^pro^ (in which the catalytic site was inactivated) nor by another EV71 protease 2A^pro^. Serial mutagenesis was performed in CstF-64, revealing that the 3C^pro^ cleavage sites are located at position 251 in the N-terminal P/G-rich domain and at multiple positions close to the C-terminus of CstF-64 (around position 500). An accumulation of unprocessed pre-mRNA and the depression of mature mRNA were observed in EV71-infected cells. An *in vitro* assay revealed the inhibition of the 3′-end pre-mRNA processing and polyadenylation in 3C^pro^-treated nuclear extract, and this impairment was rescued by adding purified recombinant CstF-64 protein. In summing up the above results, we suggest that 3C^pro^ cleavage inactivates CstF-64 and impairs the host cell polyadenylation *in vitro*, as well as in virus-infected cells. This finding is, to our knowledge, the first to demonstrate that a picornavirus protein affects the polyadenylation of host mRNA.

## Author Summary

Many viruses contain specific proteases that are essential for processing their own viral proteins. For an efficient replication within their hosts, on the other hand, viruses also utilize these proteases to cleave a number of key host proteins and hijack cellular machineries. In this study, host proteins are identified as the substrates for enterovirus 71 viral protease by adopting a proteomic strategy. Enterovirus 71 infection is highly associated with neurological complication and mortality in an era when poliovirus has been controlled by vaccination. Investigating the host substrates for enterovirus 71 protease may shed light on the viral–host interaction, ultimately providing further insight into the pathogenesis of EV71 infection. We found that 3C protease (3C^pro^) cleaved CstF-64, the latter a cleavage stimulation factor in host polyadenylation machinery. 3C^pro^ is known to enter host nucleus, whereas the replication of the virus occurs in cytoplasm. While its role in nucleus has been thought to inhibit host transcription, we found that 3C^pro^ may inhibit host-cell gene expression at host 3′-end pre-mRNA processing and polyadenylation steps. Consequently, less polyadenylated host mRNA is synthesized and more cellular resources, such as the translation factors, would be available for viral RNA expression.

## Introduction

Enterovirus 71 (EV71) belongs to the family of *Picornaviridae*, to which poliovirus also belongs. EV71 infection usually causes childhood herpagina or exanthema, which is also called hand, foot, and mouth disease (HFMD). Acute EV71 infection may also cause a severe polio-like neurological disease and significant mortality. EV71-related neurological complications include aseptic meningitis, brainstem and/or cerebellar encephalitis, acute flaccid paralysis (AFP), myocarditis, and rapid fatal pulmonary edema and hemorrhage, which have been observed during outbreaks in Taiwan, mainland China, Malaysia, Brunei, Singapore, western Australia, the United States, and Europe [1]–[10].

Numerous host machineries are affected by picornaviral infection, including host cap-dependent translation [11],[12] and transcription [13],[14]. Viral proteases are responsible for inhibitory effects. Picornaviruses typically encode two viral proteases, 2A^pro^ and 3C^pro^, which are important for viral polypeptide processing. The major catalytic sites of type I poliovirus and EV71 3C^pro^ are His40, Glu71, and Cys147 [15]–[20]. In general, enterovirus 3C proteinases cleave at Gln/Gly scissile pairs [21]. Picornaviral 3C^pro^ can enter nuclei through its precursor 3CD′ or 3CD, which contains a nuclear localization sequence (NLS) [22],[23], and can cleave some cellular transcriptional factors or regulators, such as TATA-box binding protein, p53, Histone H3 and transcription factor IIIC [13], [14], [24]–[27], offering insight into the effects of these factors or regulators on the transcriptional machinery of the host. Therefore, the identification of other cellular proteins that are cleaved by EV71 3C^pro^ in nuclei is of interest, as it may help us to understand unknown viral-host interactions during EV71 infection. In this work, a novel nuclear factor, CstF-64, that is cleaved by EV71 3C^pro^, is identified.

Poly(A) tails are important for both cellular mRNA and picornaviral mRNA. The poly(A) tail in eukaryotic cells is suggested to confer mRNA stability, promote the translational efficiency of mRNA, and the transport mRNA from the nucleus to the cytoplasm [28]–[31]. The poly(A) tail of poliovirus is critical to viral replication [32],[33]. However, poly(A) for picornaviruses is obtained differently from that for host cells. The poly(A) tail of picornaviruses is encoded by the genome, copied into a 5′ poly(U) tract in the negative sense form, then back into a poly(A) tail during the virus RNA replication cycle [34]. Polyadenylation of most eukaryotes involves a series of steps. Prior to poly(A) synthesis, a polyadenylation signal located at the 3′ end of precursor mRNA (pre-mRNA) must be recognized by multiple factors such as cleavage factors I and II (CF I and II), cleavage/polyadenylation specificity factor (CPSF) and cleavage stimulation factor (CstF) complex, which causes endonucleolytical cleavage at the site of polyadenylation [35],[36]. One of these factors, cleavage stimulation factor, 3′ pre-RNA, subunit 2, 64 kDa (CstF-64) is responsible for recognizing the second polyadenylation sequence element, a G/U-rich motif which is down-stream of the polyadenylation sites on pre-mRNA [37]. *In vitro* and *in vivo* blockage of CstF-64 impairs the 3′-end pre-mRNA processing and polyadenylation [38],[39].

This work identified eight cellular nuclear proteins as potential targets for EV71 3C^pro^ cleavage by two-dimensional (2D) electrophoresis and MALDI-TOF analysis. CstF-64, the target selected from these eight proteins, is further examined. CstF-64 in the nuclear extract was verified by western blot to be degraded upon EV71 3C^pro^ treatment. The reduction and cleavage pattern of CstF-64 in EV71-infected cells were studied. The EV71 3C^pro^ cleavage sites in CstF-64 were also mapped by performing an *in vitro* cleavage assay. The 3′-end pre-mRNA processing in host cells was monitored during EV71 infection. An *in vitro* assay was also conducted to test the effect of 3C^pro^ on the cellular 3′-end pre-mRNA processing and polyadenylation. The findings herein are the first to demonstrate that a picornaviral protease interferes with host gene expression at the machinery of polyadenylation.

## Results

### Identification of CstF-64 as Potential EV71 3C^pro^ Substrate in Nuclear Extract

**Figure 1:**
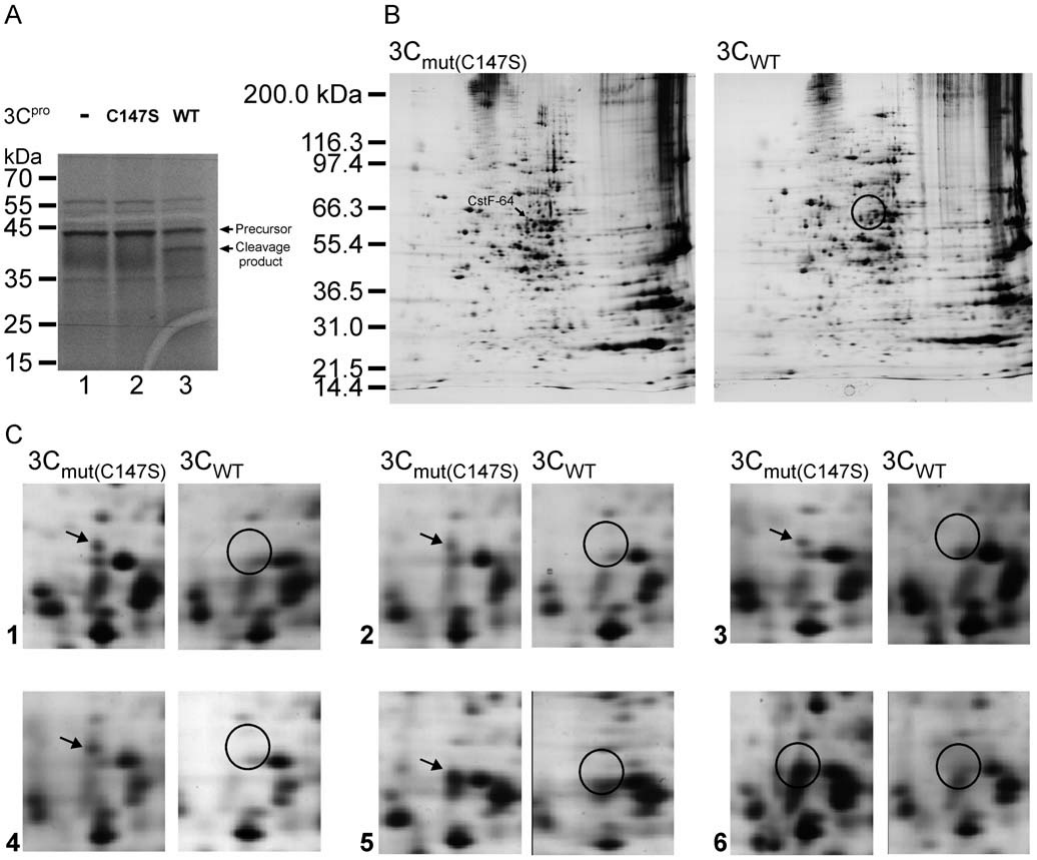
Identification of potential EV71 3C^pro^ substrates by 2D gels. (A) A [^35^S]-labeled peptide which contains part of EV71 viral polyprotein was treated with wild-type 3C^pro^(WT) or mutant 3C^pro^ (C147S). The uncleaved peptide precursor and 3C^pro^ cleavage product was indicated. (B) Full scale of 2D gels for one of the six *in vitro* cleavage experiments; left panel shows results obtained using the mutant 3C^pro^-treated nuclear extract (3C_mut_); right panel shows the results obtained using wild-type 3C^pro^-treated nuclear extract (3C_WT_). Arrows indicate location of CstF-64. (C) Six *in vitro* cleavage experiments on 2D gels. Arrows indicate location of CstF-64 protein on mutant 3C^pro^-treated nuclear extracts gels; circles indicate the corresponding regions on wild-type 3C^pro^-treated nuclear extract gels.

Picornavirus 3C^pro^ proteins can enter the nuclei of host cells [22],[23]. To identify potential substrates of EV71 3C^pro^ in nuclei, *in vitro* 3C^pro^ cleavage assay was conducted and the proteomic technique was utilized to identify the cellular targets. First, the catalytic activities of purified recombinant EV71 wild-type 3C^pro^ or mutant 3C^pro^(C147S) were verified using a [^35^S]-labeled peptide substrate, which contains a 3C^pro^ cleavage site, as described elsewhere [19]. The result shows that the wild-type, but not the C147S mutant of 3C^pro^ cleaves the viral substrate (Fig. 1A). The recombinant EV71 wild-type 3C^pro^ or mutant 3C^pro^ were then added to nuclear extracts from SF268 cells. Following incubation for 4 hours at 37°C, the reactions were subjected to 2D electrophoresis. The experiments were performed six times and the results were analyzed using PDquest 7.0 (Bio-rad). Fig. 1B presents one of these six 2D electrophoresis experiments. Proteins that appeared in the mutant 3C^pro^-treated reactants but in at least half of the amounts of the wild-type 3C^pro^-treated reactants, based on silver staining, were regarded as potential targets. From the six pairs of gels in these 2D gel experiments, eight proteins that yielded similar results at least three times were selected. These proteins were then identified by MALDI-TOF mass spectrometry and the results were summarized in Table 1. In contrast with that of mutant 3C-treated nuclear extract, CstF-64 of the wild-type 3C^pro^-treated nuclear extract was decreased to nearly undetectable levels in five individual 2D electrophoresis gel (Fig. 1C). Arrows in Figs. 1B and 1C indicate the locations of CstF-64 on 2D gels. These results suggest that CstF-64 is a potential substrate for EV71 3C^pro^.

**Table 1:** Identification of potential 3C^pro^ substrates by MALDI-TOF analysis.

No.	NCBI Accession No.	Protein name	Mw. (Da)/IP	Sequence Coverage	Total length(a.a.)
01	gi|32879899	Cleavage stimulation factor, 3′ pre-RNA, subunit 2, 64 kD (CstF-64)	61035/6.36	24%	577
02	gi|5689738	Nuclear matrix protein NMP200	55603/6.14	52%	504
03	gi|6755578	SWI/SNF related, matrix associated, actin dependent regulator of chromatin, subfamily b, member 1	44398/5.8	44%	385
04	gi|28422247	CTBP2 protein	49415/6.47	43%	445
05	gi|56550081	BUB3 budding uninhibited by benzimidazoles 3 isoform b	37330/6.36	51%	326
06	gi|47939618	Heterogeneous nuclear ribonucleoprotein A1	34273/9.2	79%	310
07	gi|5107637	Chain C, structure of the karyopherin beta2-Ran Gppnhp nuclear transport complex	24519/7.01	27%	216
08	gi|28958118	Structural maintenance of chromosomes 3	141819/6.77	33%	1217

Note: The sequence coverage for these proteins determined by MALDI-TOF analysis is also indicated. (Mw: molecular weight; IP: isoelectric point; a.a.: amino acids).

### Reduction of CstF-64 in Nuclear Extracts by Addition of 3C^pro^ *In Vitro*

**Figure 2:**
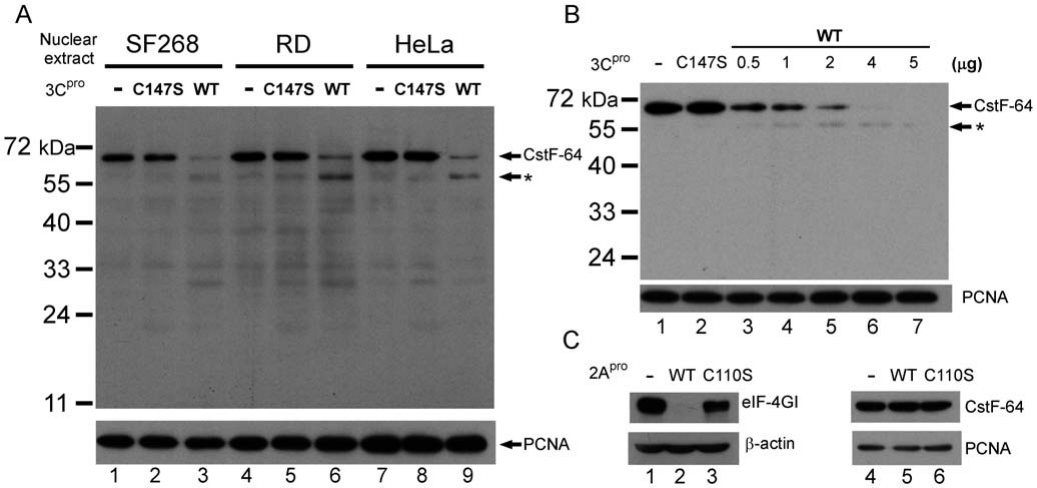
CstF-64 in 3C^pro^-treated nuclear extract. (A) CstF-64 proteins in nuclear extracts from SF268, RD and HeLa cells were detected using anti-CstF-64 antibodies. Untreated nuclear extracts (-) were loaded as controls. The amount of CstF64 in wild-type 3C^pro^ treated nuclear extracts (WT) and in mutant 3C^pro^-treated nuclear extracts (C147S) were showen. A cleavage product of 55 kDa was indicated (*). PCNA (proliferatory cell nuclear antigen) detection was employed as the loading control. (B) CstF-64 protein in RD nuclear extract treated with various quantities of 3C^pro^ - 0.5 µg , 1 µg, 2 µg , 4 µg and 5 µg (lanes 3 to 7) and in the mutant 3C-treated nuclear extract (C147S) or untreated nuclear extract (-) were shown. (C) eIF4-GI in wild-type (WT) and mutant 2A^pro^ (C110S) treated RD cell extracts were detected using specific antibody (lanes 1–3). CstF-64 in 2A^pro^ or mutant 2A^pro^-treated nuclear extract was also detected (lanes 4–6).

The reduction of CstF-64 by proteomic assay was confirmed by using an anti-CstF-64 antibody [40] to detect the level of CstF-64 in nuclear extracts from SF268 cells, which were incubated with wild-type or mutant recombinant 3C^pro^. The amount of CstF-64 in SF268 nuclear extract dramatically declined following incubation with wild-type 3C^pro^ (Fig. 2A, lane 3), and the intact form of CstF-64 was clearly detected in mutant 3C^pro^-treated nuclear extract (lane 2). One potential cleavage product of 55 kDa was also detected using CstF-64 antibody in wild-type 3C^pro^-incubated nuclear extract (lanes 3, 6 and 9, indicated by *). CstF-64 levels in nuclear extracts from other cell lines, RD and HeLa cells, also declined upon incubation with wild-type 3C^pro^ and the cleavage pattern is similar to that in SF268 nuclear extracts. (Compare lanes 6 and 9 with results for mutant 3C^pro^-treated nuclear extracts in lanes 5 and 8). The wild-type 3C^pro^-induced CstF-64 reduction in nuclear extract depends on dose of recombinant 3C^pro^ (Fig. 2B, lanes 3–7). The intensities between the full-length CstF-64 and its 55 kDa cleavage product were not the same as expected. This discrepancy is attributed to that later in the experiments, the cleavage sites were mapped to position 251 and multiple positions close to the C-terminus (around position 500) of CstF-64. The cleavage of CstF-64 at positions around 500 produce a product of 55 kDa, which contains another 3C^pro^ cleavage site at its Gln251. When position 251 was mutated (Gln to Ala), the mutant CstF-64 was cleaved into a 55 kDa product with the same intensity as that of full-length CsfF-64. Fig. 2A shows a band faster than 24 KDa (lane 2, 5 and 8), which we speculate is a cleavage product from a degraded form of CstF-64. The degraded form of CstF-64 might contain the cleavage sites for 3C^pro^ as well as the CstF-64 antibody recognition sites. Therefore, western blot analysis detected the cleaved product from the degraded form only in mutant 3C^pro^, but not in wild-type 3C^pro^-treated nuclear extract because the cleavage destroyed the antibody recognition of CstF-64. The other EV71 viral protease, 2A^pro^ was also tested to determine whether it could degrade CstF-64 *in vitro*. The catalytic activities of recombinant EV71 2A^pro^ were firstly elucidated by treatment with RD cell lysate and were verified by western blot for a known 2A^pro^ substrate, eIF-4GI, to be able to be cleaved *in vitro* (Fig. 2C, lane 1–3). The western blot for CstF-64 in the nuclear extracts that were treated by purified recombinant 2A^pro^ demonstrated the inability of 2A^pro^ to induce CstF-64 degradation *in vitro* (Fig. 2C, lane 4–6). These results indicate that viral 3C^pro^, but not 2A^pro^ is responsible for reducing CstF-64 *in vitro*.

### Reduction of CstF-64 in EV71-infected Cells

**Figure 3:**
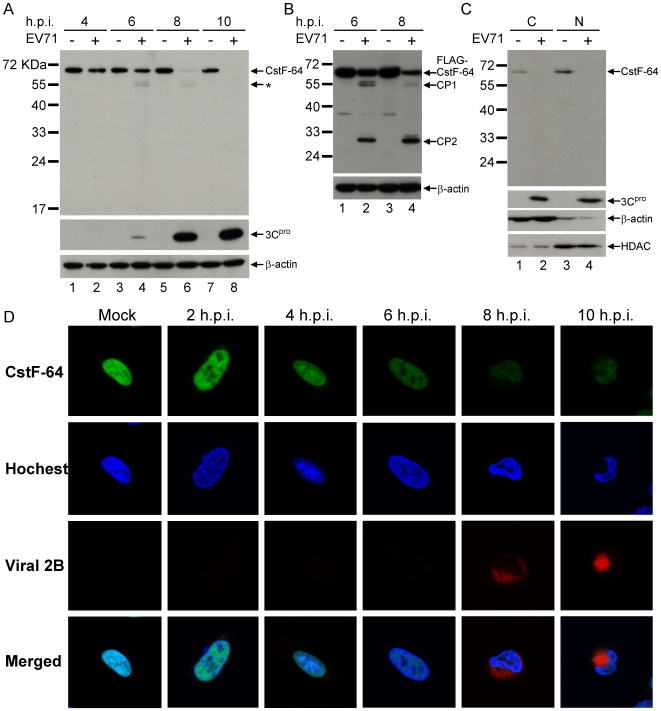
CstF-64 in EV71-infected cells. (A) After RD cells have been infected with EV71 (m.o.i. = 40), 3C^pro^ and CstF-64 protein in total cellular proteins of infected RD cells (+) or mock infected cells (-) were detected at various hours post-infection (h.p.i.) using specific antibodies. A potential cleavage product of CstF-64 was also indicated (*). The detection of β-actin was used as a loading control (B) RD cells with transient FLAG-CstF-64 overexpression were also infected with EV71 at an m.o.i. of 40 and the total cellular proteins from mock infected (-) and infected cells (+) were harvested at 6 and 8 h.p.i.. FLAG-CstF-64 was detected using FLAG-specific antibody. The other cleaved FLAG-peptides were indicated as CP1 and CP2 (C) Cytoplasmic (C) and nuclear (N) fractions from EV71-infected RD cells at 8 h.p.i. were extracted and CstF-64 or 3C^pro^ in each of the fractions were detected using specific antibodies. Detections of β-actin and histon deacetylase (HDAC) were used as cytoplasmic and nucleus protein controls. (D) The locations of CstF-64 in uninfected (Mock) or EV71-infected cells at 2, 4, 6, 8 and 10 h.p.i. were detected using specific antibody. The detection of viral 2B protein was applied as an infection-positive marker. The nuclei of cells were stained using Hoechst dye.

*In vitro* assays demonstrated that EV71 3C^pro^ can cleave CstF-64; the degradation of CstF-64 in EV71-infected cells was, therefore, examined. A time-course study was conducted to monitor the levels of CstF-64 in RD cells infected with EV71 at a multiplicity of infection (m.o.i.) of 40. Total cell lysates harvested from EV71-infected RD cells at various hours post-infection (h.p.i.) were analyzed by western blot assay. Infection reduced the amount of host CstF-64 proteins from 6 to 10 hours post-infection (h.p.i.) (Fig. 3A, lanes 4, 6 and 8). One potential CstF-64 cleavage product of 55 kDa (indicated by *) which resembles that obtained by *in vitro* cleavage, began to be detected at 6 h.p.i. (lane 4). EV71 3C^pro^ in the same infected cells was also observed at 6 h.p.i. (Fig. 3A, lane 4), and its level increased markedly from 8 to 10 h.p.i. (Fig. 3A, lane 6–8), in a manner that is related to the degradation times of CstF-64 in infected cells. The reduction of CstF-64 and the appearance of 55-kDa product were also observed in cells infected with a lower titer (m.o.i of 1) of EV71 (Fig. S1), but with a 2 hour delay. To detect other cleavage products from CstF-64, a CstF-64 that was fused with FLAG at its N-terminal was overexpressed in EV71-infected cells and detected using FLAG antibody at 6 and 8 h.p.i.. During EV71 infection, FLAG-CstF-64 was cleaved into products of size 55 kDa and 30 kDa (CP1 and CP2 in Fig. 3B). The 55 kDa products from FLAG-CstF-64 reduced at late point of EV71 infection (Fig. 3B, CP1, compare lanes 2 and 4), similar to the result from endogenous CstF-64 in EV71-infected cells (Fig. 3A, the bands marked as * in lane 4 and 6). On the other hand, another cleavage product of about 30 kDa was increased during virus infection (Fig. 3B, CP2 compare lanes 2 and 4). The 55 kDa intermediate cleavage products suggest that CstF-64 may be cleaved at more than one cleavage site in EV71-infected cells.

Picornavirus infection can relocalize numerous nuclear proteins to the cytoplasm [41]–[43]. To determine whether CstF-64 also undergoes changes in sub-cellular localization, the location of CstF-64 in EV71-infected was monitored by immunohistostaining using a confocal microscope. The result demonstrated that most CstF-64 remains in the nucleus of the infected cells at 6 to 10 h.p.i. (Fig. 3D), whereas the other nuclear factor, heterogeneous nuclear ribonucleoprotein K (hnRNP K), is redistributed to the cytoplasm (Fig. S2). Our 3C Ab was unable to be used in confocal experiment; therefore to determine whether EV71 3C^pro^ was also present in the nucleus, EV71-infected cells were fractionated into cytoplasmic and nucleus fractions. Western blot demonstrated that EV71 3C^pro^ is equally distributed across cytoplasmic and nuclear fractions (Fig. 3C, lanes 2 and 4), indicating that EV71 3C^pro^ can enter into a host nucleus during virus infection, as can other picornaviruses [44]. CstF-64 was detected to be localized primarily in the nuclei of uninfected cells (Fig. 3C, lane 3 compared to lane 1), consistent with previous findings [38] and confocal data presented herein (Fig. 3D). However, CstF-64 in both nuclei and cytoplasm were cleaved during virus infection (Fig. 3C, lanes 2 and 4).

### 3C^pro^ Cleaves CstF-64 Protein *In Vitro*

The results presented above suggest that the CstF-64 was reduced in wild-type 3C^pro^-treated nuclear extracts but not in mutant 3C^pro^-treated nuclear extracts (Fig. 2A). Therefore, 3C^pro^ is reasonably hypothesized to cause the reduction of CstF-64 by proteolytic cleavage.

**Figure 4:**
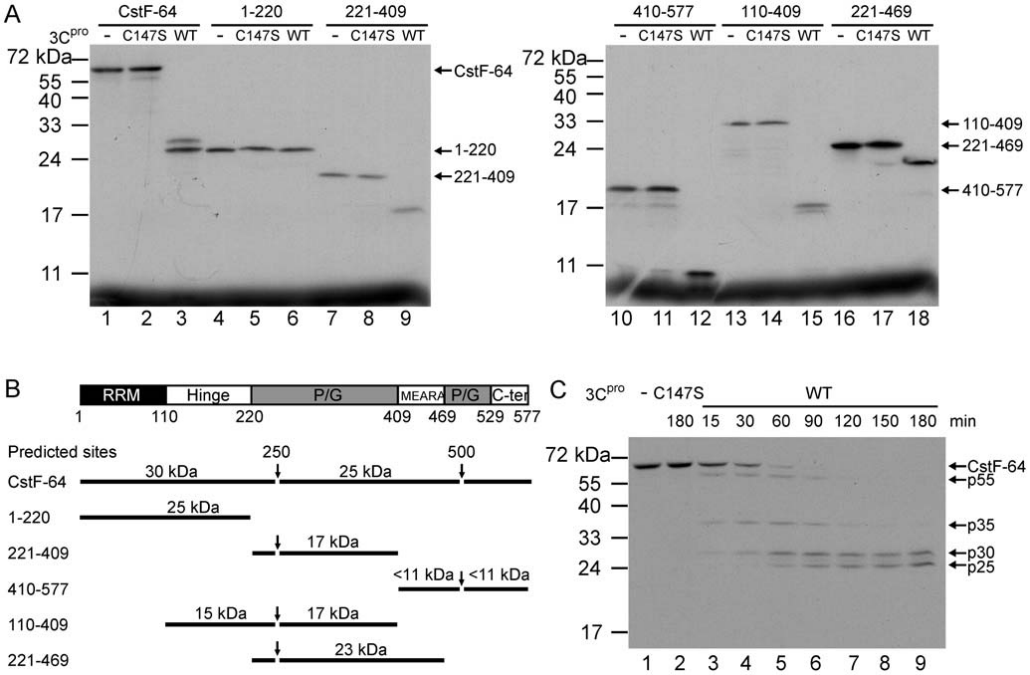
3C^pro^ cleaves recombinant CstF-64 protein *in vitro*. (A) To locate the 3C^pro^ cleavage sites on CstF-64, [^35^S]-labeled CstF-64 and numerous partial CstF-64 peptides which contain 1^st^–220^th^, 221^st^–409^th^, 410^th^–577^th^, 110^th^–409^th^, 221^st^–469^th^ amino acids of CstF-64 were generated based on the functional domains of CstF-64, including RNA recognition motif (RRM), hinge domain, Pro/Gly rich domains (P/G), MEARA sequence, and C-terminal domain (C-ter) as displayed in (B). These untreated [^35^S]-labeled peptides (-) and those were treated with wild-type 3C^pro^ (WT) and mutant 3C^pro^ (C147S) were analyzed in SDS-PAGE. The mass of cleavage products and suspected cleavage sites (arrows) are summarized in (B), indicating that the predicted 3C^pro^ cleavage sites of full-length CstF-64 are around the amino acid positions 250 and 500 of CstF-64. (C) The untreated [^35^S]-labeled CstF-64 proteins (-) and those incubated with catalytic mutant 3C protein (C147S) or wild-type 3C^pro^ (WT) with various incubation times (15, 30, 60, 90, 120, 150 and 180 minutes) were analyzed in SDS-PAGE. The cleavage products of 55 kDa (p55), 35 kDa (p35), 30 kDa (p30) and 25 kDa (p25) were also indicated.

Details of the cleavage were elucidated using a [^35^S]-labeled CstF-64 generated by *in vitro* transcription and translation (TNT) as a substrate in the *in vitro* 3C^pro^ cleavage assay. After it had incubated with recombinant 3C^pro^, [^35^S]-labeled CstF-64 can be cleaved into fragments of approximately 25 and 30 kDa (Fig. 4A, lane 3). Since the size of these two peptides do not correlate with the full-length size of CstF-64 (64 kDa compared to 25 kDa+30 kDa = 55 kDa), at least one cleavage product smaller than 10 kDa that could not be detected in PAGEs was expected to exist. To identify the 3C^pro^ cleavage sites on CstF-64, [^35^S]-labeled CstF-64 and a series of truncated CstF-64 peptides that were designed according to functional domains of CstF-64 (Fig. 4B) were generated *in vitro* by TNT for 3C^pro^ cleavage assay. Fig. 4B summarizes the size of these cleavage products, estimated from Fig. 4A. The peptides 221–409 and 410–557 were both cleaved suggesting that CstF-64 included at least two cleavage sites. The 20 kDa peptide 410–557 was cleaved into the product of 10 kDa, suggesting that a cleavage site was located close to the C-terminus of CstF-64. Peptide 1−220 (25 kDa) was not cleaved but peptides 221−409 and 110−409 were both cleaved into a product of about 17 kDa, suggesting that one cleavage site can produce a product of 30 kDa from the N-terminal of CstF-64. The cleavage of peptide 221−469 supported this conclusion. In summary, these cleavage results suggest the existence at least two potential cleavage sites around the amino acid position 250 and 500 of the CstF-64 protein.

A high amount of 3C^pro^ (5 µg) was used in above *in vitro* cleavage assay, and the incubation time was fixed at 3 hours (Fig. 4A). An attempt was made to detect the cleavage intermediates by performing *in vitro* kinetics analysis of [^35^S]-labeled CstF-64 cleavage by recombinant 3C^pro^. According to Fig. 4C, three cleavage products began to appear at 15 min. A 55 kDa product resulted from cleavage at positions around 500, while two products of 30 (front) and 35 (rear) kDa both resulted from cleavage at position around 250. A new 25 kDa product began to appear at 60 min, in addition to the three previously detected products. This 25 kDa segment is attributed to the further cleaving of either the aforementioned 35 (rear) kDa product at positions around 500 or the 55 kDa product at position around 250. As the incubation time exceeded 2 hours, only the 25 and 30 kDa products dominated and became the final cleavage products. Various concentrations of 3C^pro^ were added to the reactions (Fig. S3), and similar cleavage products were observed, as shown in Fig. 4C.

### Mapping 3C^pro^ Cleavage Sites on CstF-64 *In Vitro*

**Figure 5:**
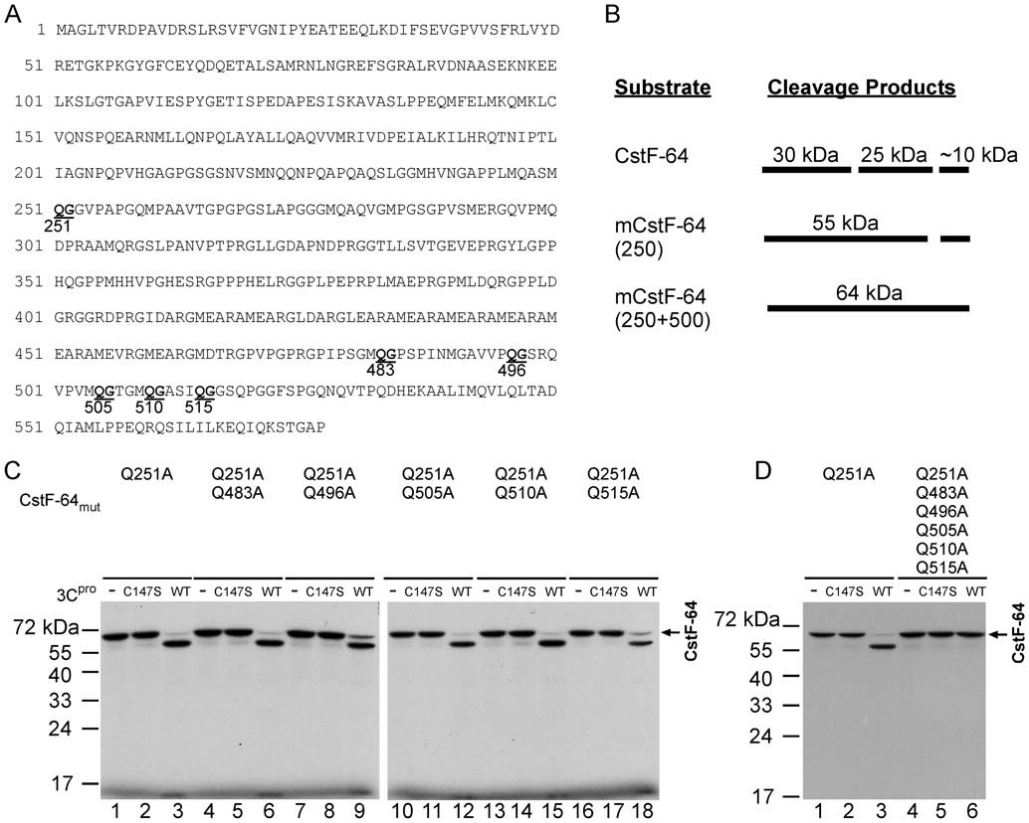
Multiple 3C^pro^ cleavage sites around the amino acid positions 250 and 500 of CstF-64 *in vitro*. (A) Gln/Gly (Q/G) junctions located around amino acid positions 250 and 500 of CstF-64, including Gln251, 483, 496, 505, 510 and 515. (B) Predicted size of 3C^pro^ cleavage products on CstF-64 with mutations at the cleavage site close to amino acid position 250 of CstF-64 (250) or at both the cleavage sites at positions 250 and 500 (250+500). (C) Wild-type 3C^pro^ (WT) or mutant 3C^pro^ (C147S)-treated [^35^S]-labeled CstF-64(Q251A) with a single Gln483, 496, 505, 510 or 515 (Q483A, Q496A, Q505A, Q510A, Q515A) mutation (D) Wild-type 3C^pro^ (WT) or mutant 3C^pro^ (C147S)-treated [^35^S]-labeled CstF-64(Q251A) or CstF-64(Q251A) with multiple mutations of Q483A, Q496A, Q505A, Q510A and Q515A.

Picornavirus 3C^pro^ generally cleaves peptides at the Gln/Gly junction [21]. Based on the cleavage results presented above, an analysis of the amino acid sequence of CstF-64 was conducted, revealing that numerous Gln/Gly junctions may have potential 3C^pro^ cleavage sites on CstF-64 (Fig. 5A). They are Gln251, corresponding to the predicted cleavage site in the region of amino acid 250, and residues Gln483, 496, 505, 510, 515 at around the amino acid position 500 on CstF-64. To determine which Gln/Gly junction on CstF-64 is the actual 3C^pro^ cleavage site, an attempt was made to produce mutant [^35^S]-labeled CstF-64 at putative Gln/X cleavage sites. Since two cleavage sites around the amino acids positions 250 and 500 of CstF-64 were estimated, cleavage at position around 250 is expected to produce a product of ∼55 KDa. However, as is predicted, a CstF-64 in which both 250 and 500 sites are mutated would be fully withstand 3C^pro^ cleavage (Fig. 5B). Following 3C^pro^-treatment, the CstF-64 with the mutation of Gln251 into Ala (Q251A) yielded a cleavage product of 55 KDa (Fig. 5C, lanes 1–3), which is consistent with the predicted product when the cleavage site around amino acid position 250 is blocked (Fig. 5B). This result suggests that the Gln251 of CstF-64 is the only 3C^pro^ cleavage site around the position 250 *in vitro*. To identify the other cleavage sites around the amino acid position 500 of CstF-64, CstF-64(Q251A) was further mutated at the Gln483, 496, 505, 510 and 515 amino acids into Ala (Q483A, Q496A, Q505A, Q510A and Q515A, respectively). The results indicate that CstF-64(Q251A) peptides with single mutations on these potential cleavage sites are not completely resistant to wild-type (WT) 3C^pro^ treatment (Fig. 5C, lanes 4–18). A mutant CstF-64(Q251A) with Q483A, Q496A, Q505A, Q510A, and Q515A is fully resistant to 3C^pro^ treatment (Fig. 5D, lanes 4–6), suggesting that the existence of multiple cleavage sites around the amino acid position 500 of CstF-64 in the five residues, Gln483, 496, 505, 510 and 515. Moreover, the cleavage pattern was examined in a mutant CstF-64 (CstF-64-5 m), in which all Gln/Gly sites around position 500 were mutated. This observation indicates that CstF-64-5 m was cleaved into two detectable products (Fig. S4): a) 30 kDa product, i.e. the N-terminal part of CstF-64 after 3C^pro^ cleaves it at Gln/Gly of position 251 and b) the 35 KDa product, i.e. the C-terminal part of CstF-64 when 3C^pro^ cleaves it at Gln/Gly of position 251, but not at Gln/Gly sites near position 500. Taken together, above results demonstrate that Gln to Ala mutations at position 251 and multiple sites around position 500 let CstF-64 become fully resistant to EV71 3C^pro^ cleavage.

### Cellular 3′ Pre-mRNA Processing Was Impaired in EV71-infected Cells

**Figure 6:**
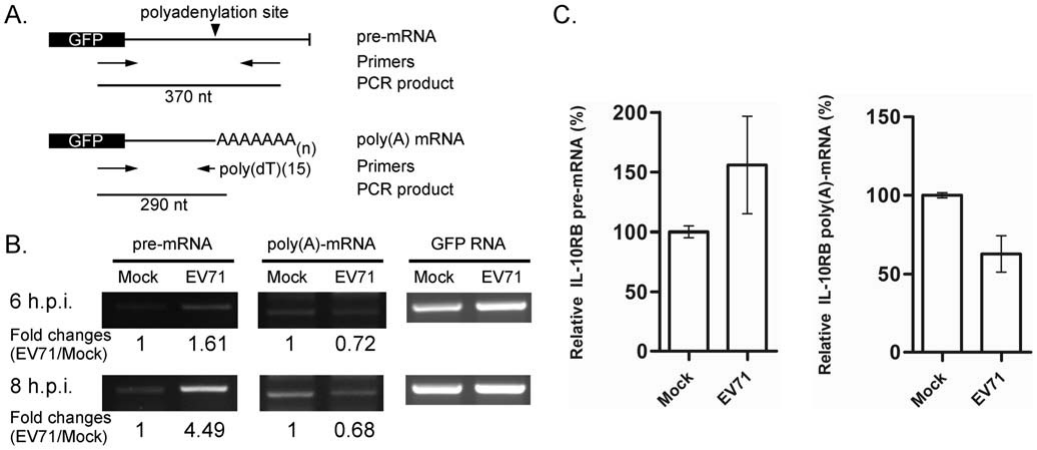
Pre-mRNA and poly(A)-mRNA in EV71-infected cells. (A) Primers that target the region downstream of the polyadenylation site and poly(A) tail sequence, were designed to detect pre-mRNA and poly(A) mRNA of GFP. (B) After plasmid pEGFP was transfected into the RD cells, cells were infected with EV71 and total RNA of infected cells was harvested at 6 and 8 h.p.i. for RT-PCR assay. RT-PCR results demonstrate the accumulation of pre-mRNA and the reduction of poly(A)-mRNA in EV71-infected cells at six and eight hours post-infection (h.p.i.). Following calibration to the total amount of GFP RNA, the fold changes from the amount of pre-mRNA and poly(A)-RNA in mock-infected cells (Mock) to that of EV71-infected cells (EV71) were calculated. (C) The endogenous pre-mRNA and poly(A)-mRNA of Interleukin-10 receptor beta (IL-10RB) in mock or EV71 infected cells at 8 h.p.i. were estimated based on real-time PCR. The relative amounts of pre-mRNA and poly(A)-mRNA were normalized by total IL-10RB RNA.

CstF-64 is an important factor for host 3′ pre-mRNA processing and RNA polyadenylation [35],[38]. Results presented above demonstrate the reduction of CstF-64 protein in EV71-infected cells; therefore, whether the machinery of host cell polyadenylation is affected by EV71 infection was further examined. Initially, the pre-mRNA processing in EV71-infected cells was assessed using an artificial exogenous expressed GFP RNA. The pEGFP plasmid contained a SV40 polyadenylation signal sequence, which was commonly used in the study of cellular mRNA processing and polyadenylation. Primers that target GFP pre-mRNA and an oligo-dT primer that targets GFP poly(A) mRNA were used in RT-PCR for detecting RNA species (pre-mRNA and mature mRNA with poly(A) tail) in EV71-infected cells (Fig. 6A). A set of primers that target the GFP coding region, which can theoretically detect both pre-mRNA and mRNA with a poly(A) tail, were also utilized as controls to demonstrate the total amount of GFP RNA in cells. RD cells were infected with EV71 after they were transfected with eGFP plasmid. Additionally, by adopting those primers designed in Fig. 6A, RT-PCR analysis was performed for semi-quantifying different GFP RNA species in these infected cells (Fig. 6B). After they were calibrated to the total amount of GFP, the results revealed the accumulation of unprocessed pre-mRNA and a reduction in polyadenylated mRNA in EV71-infected cells.

An attempt was made to identify if EV71 infection affected endogeneous pre-mRNA processing. Interlekin 10 receptor beta (IL-10RB) was selected as the target because, in an earlier study, the poly(A) mRNA of IL-10RB was found to be decreased in EV71-infected cells by cDNA microarray [45]. The relative amounts of IL-10RB pre-mRNA and poly(A) mRNA were monitored by real-time RT-PCR. According to those results, the IL-10RB pre-mRNA in EV71-infected cells decreased to 62.95% of that in mock-infected cells, which correlates with our previous micorarray results. Additionally, the relative amount of IL-10RB pre-mRNA increased to 156% of that in mock-infected cells (Fig. 6C). Above results suggest that EV71 infection impairs the CstF-64-related 3′ pre-mRNA processing mechanism.

### EV71 3C^pro^ Inhibits Cellular 3′-end Pre-mRNA Processing and Polyadenylation *In Vitro*

**Figure 7:**
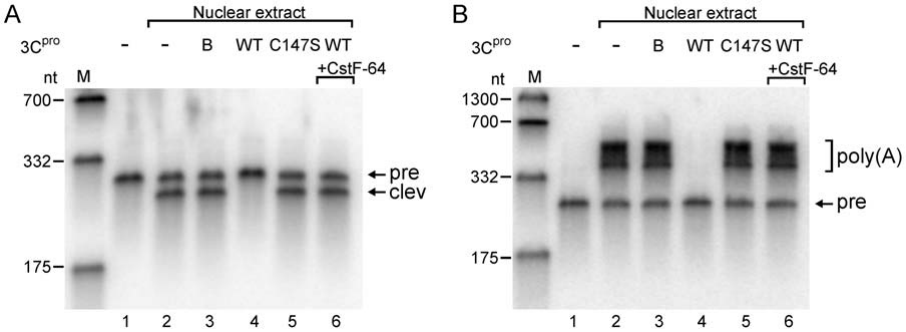
*In vitro* 3′-end pre-mRNA processing and polyadenylation of 3C^pro^-treated HeLa nuclear extract. (A) Cleavage and (B) polyadenylation of pre-RNA substrates by untreated nuclear extract (-) or nuclear extracts treated with 3C^pro^ buffer indicated as B, mutant 3C^pro^ (C147S) and wild-type 3C^pro^ (WT) were analyzed. The recruitment of recombinant CstF-64 protein after 3C^pro^ treatment (WT + CstF-64) was also tested. The un-treated RNA substrate with buffer only was used as a control. The cleavaged producted (clev) and polyadenylated RNA [poly(A)] from nuclear extract-treated pre-mRNA substrate (pre) were indicated.

The results herein suggest that EV71 3C^pro^ cleaves CstF-64, potentially inhibiting cellular pre-mRNA 3′-end formation and polyadenylation. An *in vitro* assay was conducted to test the effect of EV71 3C^pro^ on HeLa nuclear extract. The substrate employed in this assay is a capped pre-mRNA, which contains an SV40 late gene polyadenylational cleavage site [38]. A comparison with the mutant 3C^pro^-treated nuclear extracts (Fig. 7A, lane 5 and Fig. 7B, lane 5) or untreated nuclear extracts (Fig. 7A, lanes 2, 3 and Fig. 7B, lanes 2, 3) shows that the cleavage of pre-mRNA and polyadenylation proceeded efficiently. However, upon wild-type 3C^pro^ treatment, the treated nuclear extracts lost the ability to perform pre-mRNA cleavage (Fig. 7A, lane 4) and polyadenylation (Fig. 7B, lane 4). Moreover, the impairments were rescued by adding purified recombinant CstF-64 protein (Fig. 7A, lane 6 and Fig. 7B, lane 6). The results suggest that EV71 3C^pro^ targeting to CstF-64 is a factor that inhibits cellular 3′-end pre-mRNA processing and polyadenylation.

## Discussion

Picornaviral 3C^pro^ reportedly influences numerous cellular functions by cleaving various host proteins, which mechanism is important in shutting off cellular gene expression in transcriptional and translational levels [13], [14], [24]–[27]. Here we show that EV71 3C^pro^ may affect another cellular mechanism, polyadenylation, since a novel substrate, CstF-64, was identified using the proteomic approach. CstF-64 is a crucial element in cellular mRNA maturation [38]. This 64 kDa component plays a role in the CstF complex that binds to the polyadenylation signal region of pre-mRNA [37]. Our *in vitro* cleavage studies revealed that 3C^pro^ cleaves CstF-64 at amino acid position 251 and around 500 close to the C-terminal domain (Figs. 4 and 5). The *in vitro* data also demonstrated the blockage of pre-mRNA 3′-end processing and the polyadenylayion activities of the nuclear extract upon 3C^pro^-treatment (Fig. 7). The inhibitory effect of EV-71 3C^pro^ resembles that of the blockage of CstF-64 by the specific antibody [38]. The results together suggest that EV71 3C^pro^ cleaves CstF-64 and thus inhibits the 3′-end processing of cellular pre-mRNA. Our semi-quantitative RT-PCR results support this claim because the cellular 3′-end pre-mRNA processing was inhibited in EV71-infected cells (Fig. 6B). Pre-mRNA and mature poly(A) mRNA in Fig. 6B were detected individually by using two pairs of primers. Different primers have varying sensitivities, making it difficult to achieve a quantitative correlation between the increase in precursor and decrease in polyadenylated RNA. However, a quantivitive real-time RT-PCR result (Fig. 6C) provides further evidence to demonstrate that the pre-mRNA processing or polyadenylation (either an exogenous RNA or an endogenous RNA) was impaired in EV71-infected cells.

Other proteins than CstF-64 may be involved in EV71-induced inhibition of pre-mRNA processing in virus-infected cells even though the levels of poly(A) polymerase in EV71-infected cells were found to be similar to those of the mock-infected cells (Fig. S5). However, our *in vitro* results in Fig. 7 demonstrate that CstF-64 is the target factor that caused 3C^pro^ to affect 3′-end processing and polyadenylation. Adding recombinant CstF-64 can restore the function of 3C-incubated nuclear extracts on 3′-end pre-mRNA processing and polyadenylation (Lane 6 in both Fig. 7A and B).

Before the surge of the proteomic method, a substrate for a protease is commonly identified using western blot analysis. The proteomic approach has the advantages of the ability to identify multiple candidates simultaneously with no limitation by antibody availability. However, 2D electrophoresis and the silver staining are occasionally unable to detect proteins visible in western blot analysis [46]. This study identified eight novel proteins as the substrates for EV71 3C^pro^ via the proteomic approach, in which no previously known substrates for other picornaviral 3C^pro^ were available, such as TATA-box binding protein, p53, Histone H3 and transcription factor IIIC [13],[14],[26],[27]. This might be due to that silver staining and western blot differ in sensitivity. Moreover, the strategy adopted in Fig. 1 is set only in a single condition, i.e. the recombinant 3C^pro^ was incubated with nuclear extract from SF268 cells for 4 hours. More potential substrates could be identified if several interaction conditions were tested, e.g., varying in incubation time and nuclear extracts from other cell lines.

The *in vitro* cleavage assay revealed that 3C^pro^ cleaves [^35^S]-labeled CstF-64 protein into detectable products of 25 kDa and 30 kDa (Fig. 4B). However, CstF-64 in 3C^pro^-treated nuclear extract yielded 55 kDa products detected by western blot (Fig. 2). A product similar to that of 55 kDa was also present in EV71-infected cells (Fig. 3A). The size of the 55 kDa products above is correlated to that of the 3C^pro^ cleavage product when the cleavage site of the amino acid position 251 on CstF-64 is mutated (Fig. 5C). The detection of FLAG in FLAG-CstF-64 overexpressed cells revealed that the proportion of 30 kDa increased and that of 55 kDa declined upon EV71-infection (Fig. 3B). A FLAG-CstF-64 with Q251A mutation was transfected into EV71-infected cells and the results indicated the accumulation of 55 kDa products but no 30 KDa product was detected (data not shown), suggesting that the 55 kDa component was an intermediate cleavage product when 3C^pro^ cleaved CstF-64 at an amino acid position ∼500. The production of multiple 55 kDa bands in infected cells (Figs. 3A and B) was also observed, explaining the multiple cleavage sites around the amino acid position 500 of CstF-64. In addition, the results of *in vitro* kinetics of CstF-64 cleavage by 3C^pro^ (Fig. 4C and Fig. S3) showed a cleavage intermediate of 55 kDa. In summary, these results showed the similarity between the cleavage patterns obtained in the *in vitro* and *in vivo* assays suggests that 3C^pro^ may have reduced CstF-64 levels via proteolytic activity in infected cells.

Single mutation at position around 500 in CstF-64 still made it susceptible to EV71 3C^pro^ cleavage, while multiple mutations cause CstF-64 become resistant to the cleavage. Analytical results indicate the C-terminal of CstF-64 may contain multiple cleavage sites; otherwise, these mutations could lead to a loss of exposure of the cleavage site.

This study also attempted to examine whether C147S mutation in EV71 impairs the viral inhibition of cellular pre-mRNA processing and polyadenylation. However, C147S mutation rendered EV71 lethal. Alternatively, transcripts derived from EV71 infectious clones, i.e. wild-type versus C147S mutant, were transfected into cells to examine how they affect cellular pre-mRNA processing and polyadenylation. In wild-type transcript -transfected cells, CstF-64 was cleaved and cellular pre-mRNA processing was inhibited (data not shown), the same phenomenon was observed in EV71 virus-infected cells. However, in C147S mutant transcript -transfected cells, no cleavage occurred in CstF-64 and no effect on cellular pre-mRNA processing was observed, which is similar to the results in mock-infected cells. Still, no detectable viral protein (mature 3C^pro^) was found. We believe that phenomenon is owing to that C147S mutation destroys the proteolytic activity of 3C^pro^ and inhibits viral protein processing and maturation. Therefore, we can not conclude that the C147S mutant in EV71-infected cells impairs the viral inhibition of cellular pre-mRNA processing.

CstF-64 reportedly contains at its N-terminal an RNA-binding domain and a hinge domain, which are responsible for the reorganization of RNA and its binding to CstF-77 or other factors [47]–[50]. No 3C^pro^ cleavage was detected in this region (1–220), suggesting that the cleaved CstF-64 maintains functions RNA binding and interaction with CstF-77 (Fig. 4B). However, the Pro/Gly-rich domains of the C-terminal region of CstF-64 form an extended α-helix structure and are suspected to be able to interact with several transcription factors [47], including binding of the transcription factor PC4 via the 100 amino acids of C-terminal CstF-64 [51]. One study noted that the truncation of the C-terminal domain (529–577) of CstF-64 inhibited cellular 3′ pre-mRNA processing [52]. Accordingly, multiple cleavage by 3C^pro^ in C-terminal domain (around 500^th^ a.a.) of CstF-64 is expected to destroy the cellular pre-mRNA processing functions of CstF-64.

The RNA virus-induced inhibition of 3′-end pre-mRNA processing has been observed in the influenza A virus. NS1A of influenza A reportedly inhibits the cellular 3′-end pre-mRNA processing by interacting with CPSF-30 which is within the same machinery co-factor as CstF-64 [53]. Similar to those in EV71-infected cells, the inhibition of the cellular 3′-end pre-mRNA processing by influenza A virus causes the loss of poly(A) mRNA and the accumulation of un-poly(A) pre-mRNA. This inhibitory effect of NS1A has also been suggested to be involved in the countering of cellular antiviral responses, including virus-induced interferon β production [54]. The capacity of NS1A to bind to CPSF-30 may also be crucial to viral replication because of high interferon-β mRNA production in NS1A mutant virus-infected cells [55].

Picornaviruses influence host-cell gene expression by the inhibition of cellular transcription and cap-dependent mRNA translation [11]–[13]. However, several cellular genes could escape the inhibition of gene expression by picornavirus infection. Our previous cDNA microarray analysis for total cellular RNA demonstrated that the level of some RNAs, related to chemokines, protein degradation, complement proteins and proapoptosis proteins increased upon EV-71 infection [45], suggesting leakage from the inhibition of transcription by EV71. Translations of of c-myc, Bip, and eIF4G mRNA have been found to be increased in poliovirus-infected cells as the cap-dependent translation shuts down, because of the presence of internal ribosome entry sites (IRES) [56]–[58]. This work proposes a novel mechanism by which picornavirus inhibits cellular gene expression in the 3′-end pre-mRNA processing step, which would probably inhibit polyadenylation of the surviving host RNAs. It has been reported that the poly(A) tail of poliovirus is essential to viral replication [32],[33], which suggests several host poly(A)-associated factors are important for viral growth. Inhibition of cellular poly(A) RNA synthesis may provide yet an additional advantage for virus replication.

In conclusion, a novel mechanism by which picornavirus inhibits cellular function was identified. CstF-64 was identified as an EV71 3C^pro^ substrate. The 3C^pro^ cleavage sites were mapped onto amino acid position 251 and the C-terminal region of CstF-64. The cleavage of CstF-64 impairs the cellular 3′-end pre-mRNA processing and polyadenylaytion. EV71 utilizes 3C^pro^ to interfere polyadenylation of host cellular RNA; however, its own poly(A) synthesis is not affected since picornaviral poly(A) tail is genetically encoded [32],[33].

## Materials and Methods

### Manipulation of cells and viruses

RD and SF268 cells were maintained in DMEM that contained 10% FBS (Glibco) at 37°C. Cells were split in a ratio of 1∶10 in fresh medium every three days. After the RD cells had grown to 80% confluence, they were washed in PBS. EV71/4643/TW viruses with a multiplicity of infection (m.o.i.) of 40 were inoculated into the cells in DMEM without FBS at room temperature for one hour to infect the cells with the virus. Following one hour of incubation, viruses and the medium were replaced by DMEM that contained 2% fetal bovine serum. Infected cells were cultured at 35°C.

### Plasmids and constructions

pET-23-EV71-3C and pET-23-EV71-m3C-C147S plasmid constructs, which contain wild-type 3C^pro^ or mutant 3C^pro^ (with Cys147 replaced by Ser) cDNA in pET-23a(+) vector for recombinant EV71 3C^pro^ production have been constructed elsewhere [19]. For CstF-64 recombinant production, cDNA of CstF-64 was cloned from the Cst-64 coding region that contained the plasmid, pZ64–18, which was a gift from Dr. James L. Manely, and then inserted into vector pET23a(+) using *Eco*RV and *Not*I. For the production of [^35^S]-labeled substrates by TNT assay, full-length and partial cDNA of CstF-64 were cloned and inserted into pcDNA3.1(+) using *Eco*RV and *Not*I. For the production of FLAG-CstF-64 in transfected cells, cDNA of CstF-64 was cloned into pFLAG-CMV-2 using *Eco*RV and *Not*I. The mutant CstF-64 construct was produced using a QuikChange Site-Directed Mutagenesis Kit (Stratagene). Plasmid pG3SVL-A, which contained sequence -142 to +138 of SV40 late gene poly(A) cleavage sites was kindly provided by Dr. James L. Manely and Dr. Yoshio Takagaki, for the *in vitro* 3′-end pre-mRNA processing and polyadenylation assay.

### Western blot analysis

PVDF membrane was blocked wih Tris-buffered saline/0.1% (vol/vol) Tween 20 that contained 5% non-fat dry milk and probed with the indicated antibody. Antibodies against CstF-64 (1∶200; from Dr. Clinton C. MacDonald or 1∶2000; Santa Cruz), FLAG (1∶2000; SIGMA), PCNA (1∶2000; Santa Cruz), HDAC (1∶2000; Santa Cruz),Poly(A) polymerase (1∶2000; Santa Cruz), actin (1∶4000; Chemicon) and EV71 3C^pro^ monoclonal antibody which generated from recombinant 3C^pro^ protein by our lab (1∶50) were used. Following washing, the membranes were incubated with HRP-conjugated anti-mouse or HRP-conjugated anti-rabbit (1∶2000). HRP was detected using a Lighting Chemiluminescence reagent (Amersham Pharmacia).

### Preparation of nuclear extracts

To perform the cleavage assay in a cell-free system, SF268, HeLa or RD cells were washed in PBS and scraped out. Packed cells were re-suspended in hypotonic buffer (10 mM HEPES pH 7.9, 1.5 mM MgCl_2_, 10 mM KCl and 0.5 mM DTT) and homogenized using a 25G needle. Homogenized cell fragments were centrifuged at 3300 g to remove cytoplasmic proteins. The pellet was washed twice in hypotonic buffer and the soluble nuclear proteins were extracted by adding buffer with a graded conc. of KCl (20 mM HEPES, 25% glycerol, 1.5 mM MgCl_2,_ 0.2 mM EDTA, 0.5 mM DTT, 0.02 to 1.6 M KCl at pH = 7.9). After centrifuged at 25000 g, the extracted nuclear proteins in supernatant were harvested and dialyzed in buffer (20 m M HEPES , 20% glycerol, 100 mM KCl,0.2 mM EDTA and 0.5 mM DTT at pH = 7.9) and then stored at −80°C.

Commercial reagents for nuclear extraction (CMN compartment protein extraction kit; Biochain) were applied to prepare the nuclear extract from EV71-infected cells.

Commercial HeLa nuclear extract (Santa Cruz) was used in *in vitro* 3′-end pre-mRNA processing and polyadenylation assay.

### Preparation and purification of recombinant protein

To produce recombinant 3C^pro^ and CstF-64 proteins, plasmid pET-23-EV71-3C, pET-23-EV71-m3C-C147S and pET-23-CstF-64, were introduced into competent *E. coli* BL21 (DE3 pLysS) and protein expression was induced using 40 µM isopropyl b-D-thiogalactopyranoside. 3C-His fusion proteins were purified using a HiTrap kit (Pharmacia).

To produce [^35^S]-labeled protein, TNT Coupled Reticulocyte Lysate Systems (Promega) was used in the *in vitro* transcription and translation reaction.

### *In vitro* protease cleavage assay

In the proteomic approach to screening potential 3C^pro^ substrates of, 30 µg of 3C^pro^ was incubated with 250 µg nuclear extracts in digestion buffer (20 mM HEPES, 20% glycerol and 100 mM KCl at pH = 7.9) with total volume of 100 µl at 37°C for four hours. In western blot assay, 10 µg of 3C^pro^ was incubated with 50 µg nuclear extract under the aforementioned conditions. To cleave recombinant CstF-64, 10 µg recombinant CstF-64 proteins interacted with 10 µg of 3C^pro^ for four hours in the same buffer as in the earlier experiments. To cleave the [^35^S]-labeled substrate, 4 µl of labeled protein from one TNT assay reaction was incubated with 5 µg of 3C^pro^ in buffer (20 mM HEPES, 20% glycerol and 100 mM KCl at pH = 7.9) with a total volume of 15 µL at 37°C for two hours.

### Two-dimensional electrophoresis and protein identification

2D gel electrophoresis was conducted using the IPGphor system (Amersham Biosciences). After the nuclear extracts had incubated with 3C^pro^ and precipitated using 20% trichloroacetic acid (TCA), the sample was dissolved in 8 M Urea, 2% CHAPS, 0.5% IPG buffer (Amersham Bioscience) and bromophenol blue. These samples were loaded on a cup-loading system (Amersham Biosciences). Isoelectric focusing was performed at 45,000 Vh in a stepwise fashion (1500 V, gradient for 2 hours; 4000 V, gradient for 3500 V.hours; 8000 V, step-on-hold until the end) in Immobiline Drystrip IEF gels with the range of pH 3–10 (Amersham Biosciences). For SDS-PAGE, the IEF gels were loaded on gels with graded acryamide conc. of 9% to 16%. Proteins on 2D electrophoresis gels were visualized by silver staining and the results were scanned as TIF files. Protein spots on scanned gels were analyzed using PDquest v7.0 (Bio-rad) software. Six pairs of gels with a similarity of over 60% were selected for further analysis. Spots that appeared in the mutant 3C^pro^-treated nuclear extracts disappeared or became at least 50% smaller upon silver staining of the wild-type 3C^pro^-treated nuclear extracts were selected as the potential target substrates for wild-type 3C^pro^. Following this selection strategy, proteins that yielded similar results to each other more than three times were identified by in-gel digestion and analyzed by Bruker Ultraflex MALDI-TOF mass spectrometry. Mass lists were performed peptide mass fingerprinting by Biotool 2.0 software and the algorithm of Masscot (http://www.matrixscience.com).

### Confocal imaging

RD cells were seeded onto 22 mm diameter coverslips to 80% confluence and were infected with enterovirus 71 (strain 4643/TW/1998) at a multiplicity of infection of 40. At each time point, the culture medium was removed and the cells were washed with PBS, and fixed with 3.7% formaldehyde for 20 min at room temperature. The cells were then washed with PBS and permeabilized using 0.3% Triton X-100 for 5 min at room temperature. Cells were washed once using PBS containing 2% FBS, incubated in blocking solution for 1 hr at room temperature. In CstF-64, hnRNP K and EV71 2B immunostaining, cells were incubated with anti-CstF-64 (diluted 1∶100; Santa Cruz), anti-EV71 2B (diluted 1∶200; Provided by Dr. Jim-Tong Horng) for 8 hours at 4°C or anti-hnRNP K (diluted 1∶200; Santa Cruz) for 1.5 hr at room temperature. After they had been washed three times with PBS, coverslips were incubated with FITC-conjugated goat anti-mouse IgG or goat anti-rabbit lgG, and Alexa Fluor 568 goat anti-rat IgG (Invitrogen) for 1 hr at room temperature. The coverslips were then washed once with PBS; treated with nuclear stain Hoechst 33258 (diluted 1∶500) for 15 min; washed three times with PBS, and mounted on glass slides with mounting fluid (75% glycerol in PBS). The images were obtained under a confocal laser-scanning microscope (Zeiss; LSM 510 NLO).

### Detection of EGFP pre-mRNA and mRNA in EV71-infected cells

After they had been transfected with pEGFP-N1 for 1 hour, RD cells were infected with EV71 virus. The total RNA of these infected cells were isolated by TRIZOL Reagent (Invitrogen) and purified by phenol/chloroform extraction. To detect EGFP pre-mRNA, a set of primers 5′- CCGGAATTCTGAGCAAAGACCCCAACGAG -3′ and 5′- CCCAAGCTTAAAATATTAACGCTTACAAT-3′, which target the sequence that was removed from pre-mRNA following polyadenylation was used. To detect poly(A) EGFP mRNA, a set of primers 5′- CCGGAATTCTGAGCAAAGACCCCAACGAG -3′ and 5′- TTTTTTTTTTTTTTTGCAGT-3′, which target the poly(A) tail and polyadenylation site of EGFP mRNA, were used. To detect total EGFP RNA, a set of primers 5′-ATGGTGAGCAAGGGCGAGGA-3′ and 5′-CTTGTACAGCTCGTCCATGC-3′, which target the coding region of EGFP was used. The RT-PCR products were placed in 2% agarose gel with ethidium bromide, and semi-quantified using software FUJIFILM Science Lab 2005 (Fuji).

### *In vitro* 3′-end pre-mRNA processing and polyadenylation assay

Capped RNA substrate with a poly(A) signal of the SV40 late gene was synthesized using a Megascript SP6 kit (Ambion) and Cap analog (Ambion). Plasmid pG3SVL-6, flanked by *Dra*I, was utilized as the template in the *in vitro* transcription reaction. The synthesized capped RNA substrate was purified by phenol/chloroform extraction before it was used in *in vitro* 3′-end pre-mRNA processing and polyadenylation assay.

The nuclear extract was treated with recombinant 3C^pro^ at 37°C for two hours before it was used in 3′ pre-mRNA processing and polyadenylation assay. After the treatment, the 3C^pro^ activity was stopped by adding protease inhibit cocktail (Roche). The volume of each 3′ pre-mRNA processing reaction with 20 µl contained 25% (v/v) nuclear extract (25 µg in total), and 2 ng of RNA subtrates with added 1 mM EDTA, 0.5 mM dATP, 10 mM creatine phosphate, 2.5% (v/v) of polyvinyl alcohol. In *in vitro* polyadenylation, dATP was replaced by ATP, and the conc. of EDTA declined to 0.08 mM with the addition of 1 mM MgCl_2_. 4 µg of recombinant CstF-64 was added into one of the 3C^pro^-treated reaction. After incubation for two hours at 37°C, the assayed RNA was purified phenol/chloroform extraction. The purified RNA was analyzed in 1.5% agarose gel and denatured using formaldehyde.

### Real-time RT-PCR

Real-time RT-PCR was performed to detect levels of IL-10RB RNA. The High Capacity cDNA Reverse Transcription Kit (Applied Biosystems) was applied for reverse transcription. To differentiate pre-mRNA and polyadenylated IL-10RB RNA, different primers were used in reverse transcription step. A specific primer 5′-CTGAGAGCTCCCAGATGACTGA-3′ that targets the region that contains polyadenylation cleavage signal on IL-10RB was used to detect the pre-mRNA. On the other hand, an oligo-d(T) was used as the primer to detect poly(A)-mRNA. For total mRNA (including pre-mRNA and poly(A)-mRNA), a random hexomer was used an the primer in reverse transcription step. For PCR step, 5′-GAGGGATCAGGGCAGCAA-3′ and 5′-CAGGGTCTGGGAGTTCTAGATGTG-3′ designed by Primer Express software (Applied Biosystems) targeting IL-10RB coding region were used. The PCR reaction was performed on 7500 Real-Time PCR System (Applied Biosystem) using SYBR Green Core Kit (Applied Biosystem).

## Supporting Information

Figure S1 CstF-64 in EV71-infected cell of m.o.i. of 1. After RD cells were infected with EV71 (m.o.i. = 1) and CstF-64 protein in total cellular protein of mock infected RD cells (Mock) or EV71-infected cells at various hours post-infection (h.p.i) were detected. The cleavage product of 55 kDa is also denoted (*).
(2.78 MB TIF)

Figure S2 The location of hnRNP K in EV71-infected cells. HnRNP K in uninfected (Mock) or EV71-infected cells at 2, 4, 6, 8 and 10 h.p.i. were detected using specific antibody. The detection of viral 2B protein was applied as an infection-positive marker. The nuclei of cells were stained using Hoechst dye.
(1.47 MB TIF)

Figure S3 CstF-64 cleaved by various amounts of recombinant 3C^pro^. [^35^S]-labeled CstF-64 proteins were treated with catalytic mutant 3C (C147S) or various amounts (0.1, 0.2, 0.5, 1.0, 1.5, 2.0, 5.0 µg) of wild-type 3C^pro^ (WT). The full-length CstF-64 (CstF-64) and the cleavage products of 55 kDa (p55), 35 kDa (p35), 30 kDa (p30) and 25 kDa (p25) are denoted.
(1.78 MB TIF)

Figure S4 The 3C^pro^ cleavage of CstF-64 with mutantion at Gln251 or position 500. [^35^S]-labeled wild-type CstF-64 (WT) or mutant CstF-64 at Gln251 (Q251A) or position 500 (Q483A Q496A Q505A Q510A Q515A) were untreated (-) or treated with wild-type 3Cpro (WT) and mutant 3C protein (C147S). The full-length of CstF-64 (CstF-64) and cleavage products of 55 kDa (p55), 35 kDa (p35), 30 kDa (p30) and 25 kDa (p25) are denoted.
(2.73 MB TIF)

Figure S5 CstF-64 and poly(A) polymerase in EV71-infected cells. CstF-64, poly(A) polymerase and 3C^pro^ in mock-infected (Mock) or EV71-infected cells at 6 and 8 hours post-infection (6 and 8 h.p.i.) were detected. The cleavage product of 55 kDa from CstF-64 is denoted as *. The β-actin was used as a loading control.
(3.90 MB TIF)
