# Supplementary figures and images for: Enterovirus 71 3C Protease Cleaves a Novel Target CstF-64 and Inhibits Cellular Polyadenylation
Source: PLoS Pathog. 2009 Sep 25;5(9):e1000593. doi: 10.1371/journal.ppat.1000593 (PMC2742901; doi:10.1371/journal.ppat.1000593)

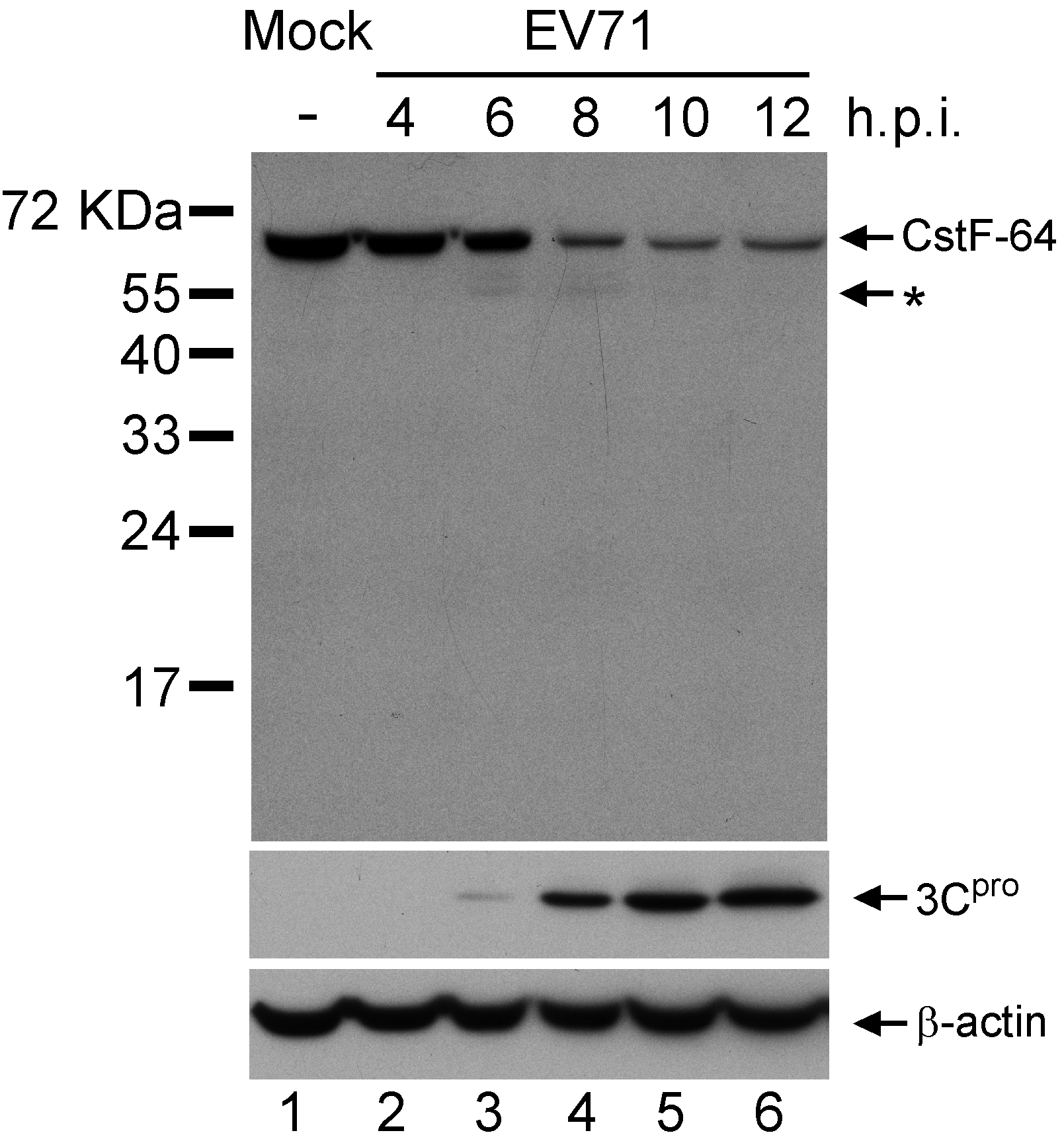

Supplement: Figure S1 — CstF-64 in EV71-infected cell of m.o.i. of 1. After RD cells were infected with EV71 (m.o.i. = 1) and CstF-64 protein in total cellular protein of mock infected RD cells (Mock) or EV71-infected cells at various hours post-infection (h.p.i) were detected. The cleavage product of 55 kDa is also denoted (*). (2.78 MB TIF) [file ppat.1000593.s001.tif]

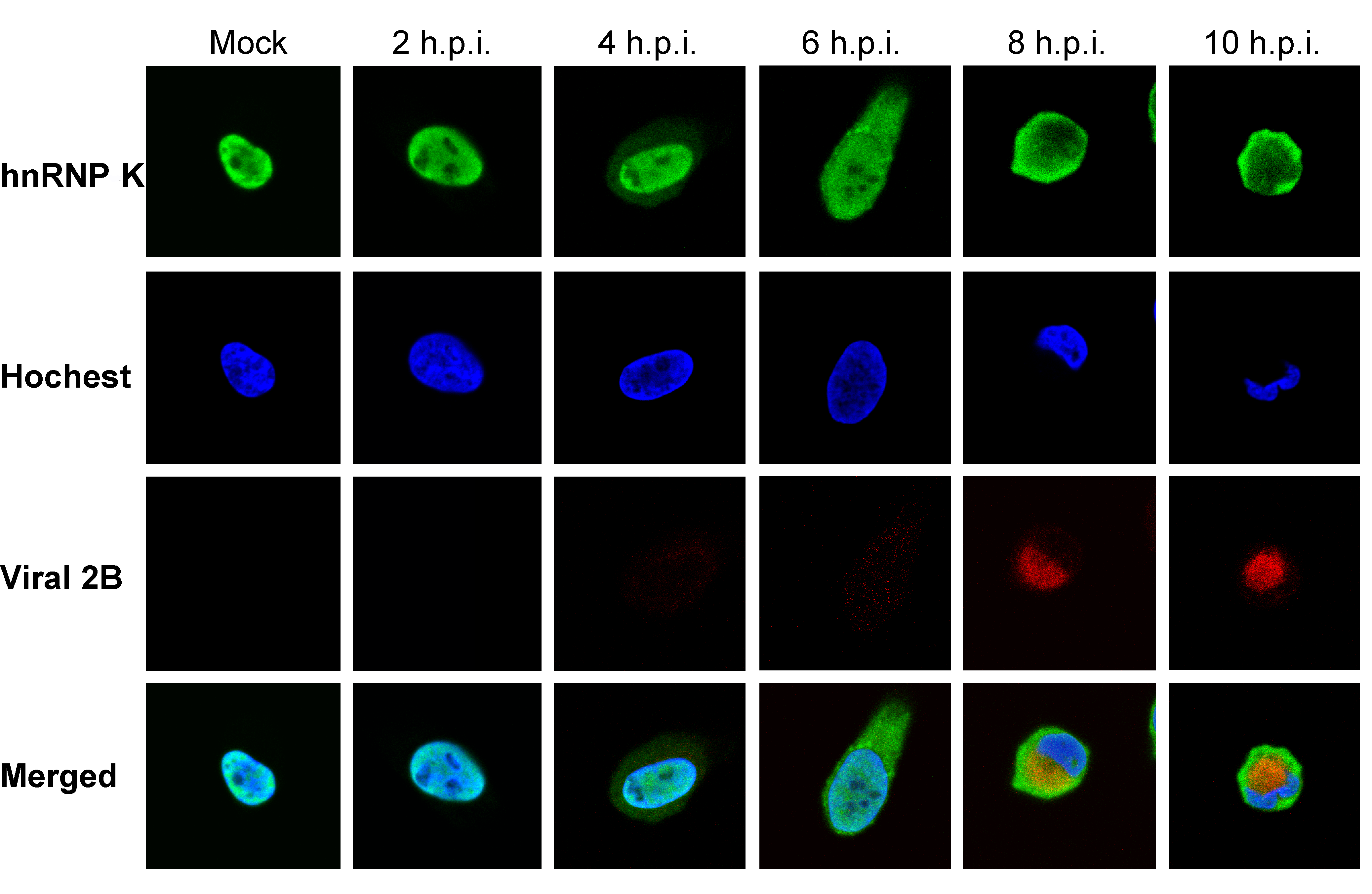

Supplement: Figure S2 — The location of hnRNP K in EV71-infected cells. HnRNP K in uninfected (Mock) or EV71-infected cells at 2, 4, 6, 8 and 10 h.p.i. were detected using specific antibody. The detection of viral 2B protein was applied as an infection-positive marker. The nuclei of cells were stained using Hoechst dye. (1.47 MB TIF) [file ppat.1000593.s002.tif]

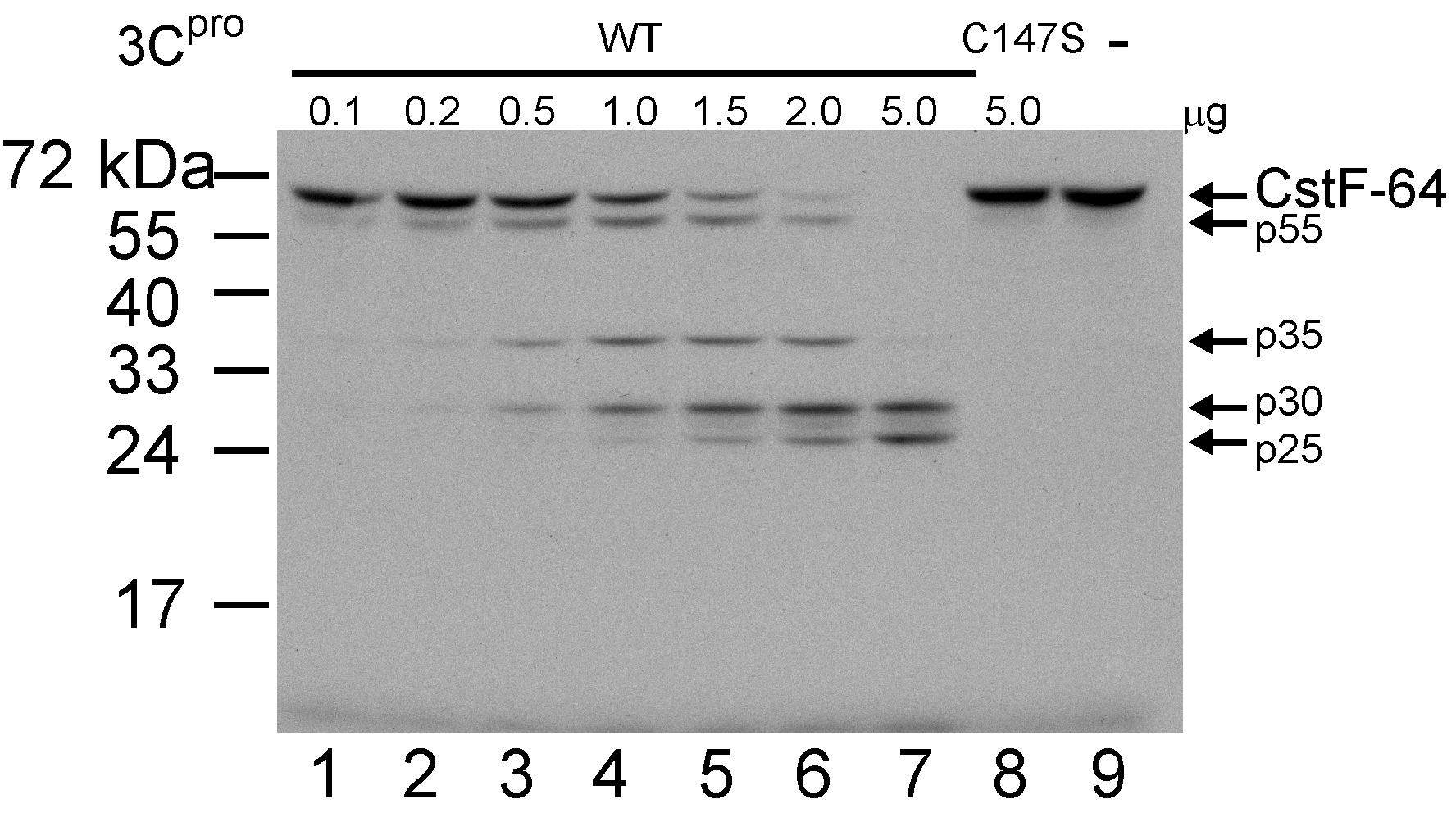

Supplement: Figure S3 — CstF-64 cleaved by various amounts of recombinant 3Cpro. [35S]-labeled CstF-64 proteins were treated with catalytic mutant 3C (C147S) or various amounts (0.1, 0.2, 0.5, 1.0, 1.5, 2.0, 5.0 µg) of wild-type 3Cpro (WT). The full-length CstF-64 (CstF-64) and the cleavage products of 55 kDa (p55), 35 kDa (p35), 30 kDa (p30) and 25 kDa (p25) are denoted. (1.78 MB TIF) [file ppat.1000593.s003.tif]

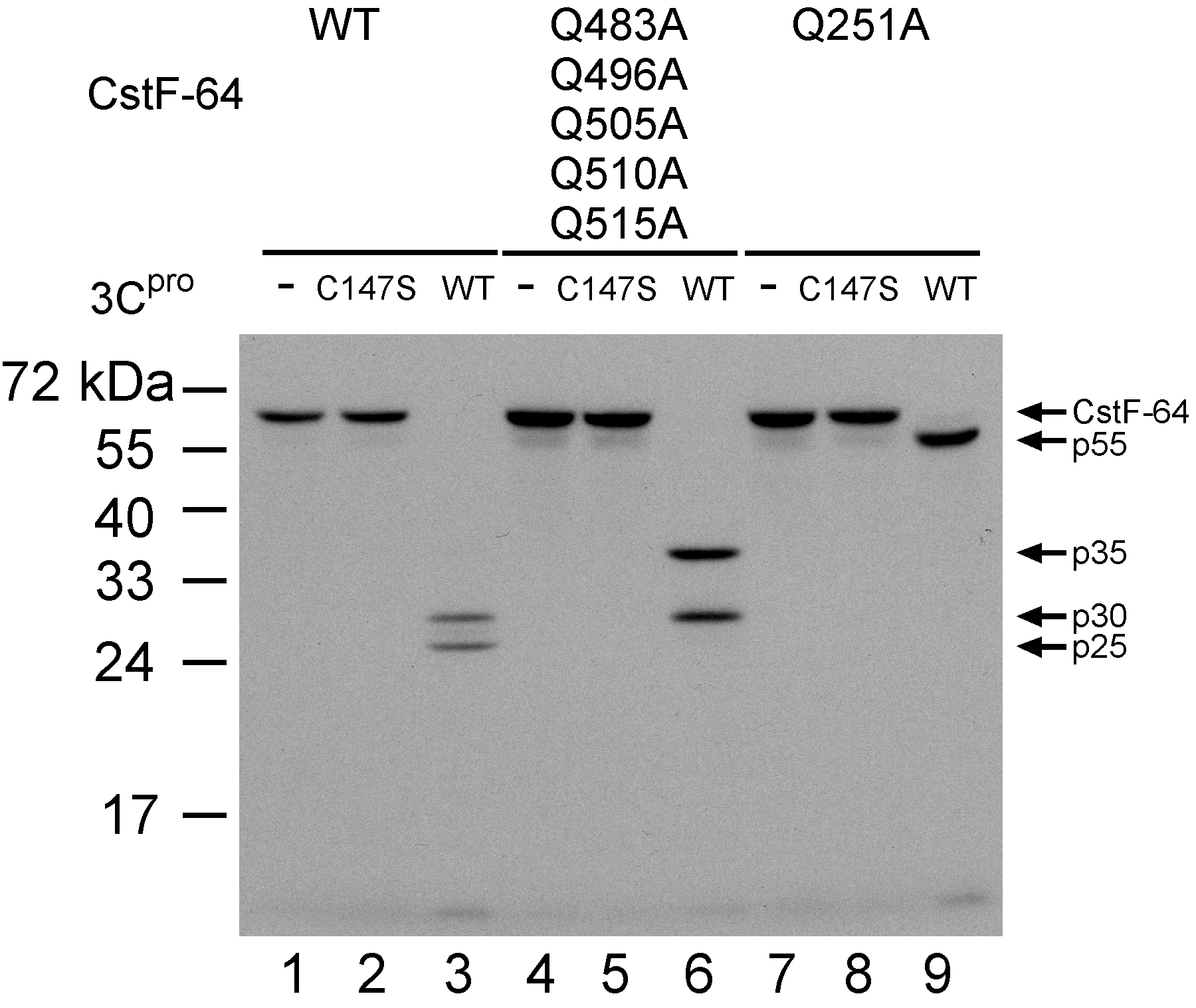

Supplement: Figure S4 — The 3Cpro cleavage of CstF-64 with mutantion at Gln251 or position 500. [35S]-labeled wild-type CstF-64 (WT) or mutant CstF-64 at Gln251 (Q251A) or position 500 (Q483A Q496A Q505A Q510A Q515A) were untreated (-) or treated with wild-type 3Cpro (WT) and mutant 3C protein (C147S). The full-length of CstF-64 (CstF-64) and cleavage products of 55 kDa (p55), 35 kDa (p35), 30 kDa (p30) and 25 kDa (p25) are denoted. (2.73 MB TIF) [file ppat.1000593.s004.tif]

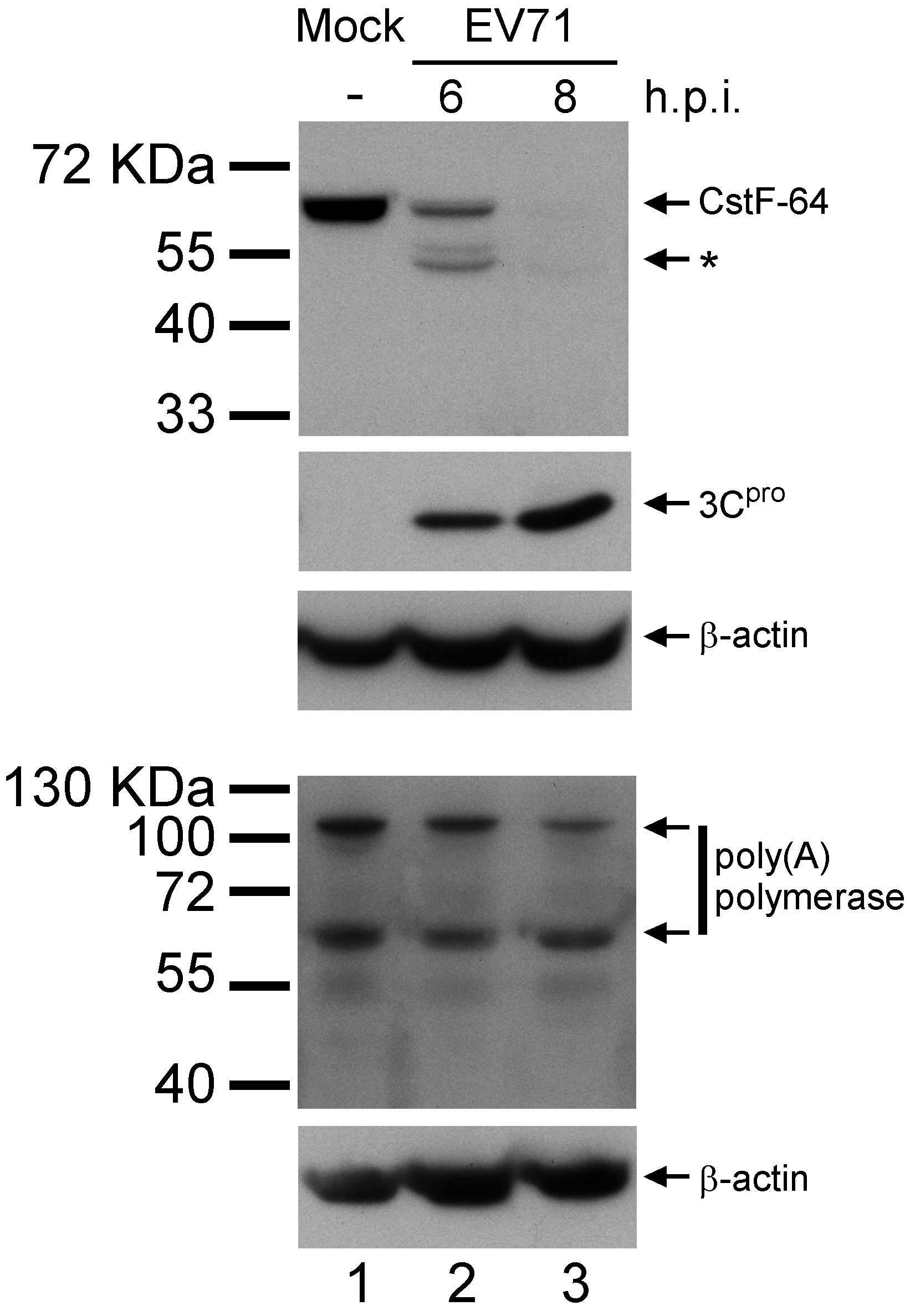

Supplement: Figure S5 — CstF-64 and poly(A) polymerase in EV71-infected cells. CstF-64, poly(A) polymerase and 3Cpro in mock-infected (Mock) or EV71-infected cells at 6 and 8 hours post-infection (6 and 8 h.p.i.) were detected. The cleavage product of 55 kDa from CstF-64 is denoted as *. The β-actin was used as a loading control. (3.90 MB TIF) [file ppat.1000593.s005.tif]
